# *DCTN1* gene analysis in Chinese patients with sporadic amyotrophic lateral sclerosis

**DOI:** 10.1371/journal.pone.0182572

**Published:** 2017-08-08

**Authors:** Xiangyi Liu, Lipeng Yang, Lu Tang, Lu Chen, Xiaolu Liu, Dongsheng Fan

**Affiliations:** Department of Neurology, Peking University Third Hospital, Beijing, China; Institute of Health Science, CHINA

## Abstract

Amyotrophic lateral sclerosis (ALS) is a fatal neurodegenerative disorder. Missense mutations of *DCTN1* have been identified as a possible genetic risk factor for ALS. Here, we tested the *DCTN1* protein-coding exons in 510 sporadic ALS patients in whom *SOD1*, *TARDBP*, *FUS*, and *C9orf72* genes were screened before. Polymerase chain reaction and Sanger sequencing were used for mutation discovery. The results revealed two rare heterozygous missense variants, c.1867C>T (p.R623W) and c.2798C>T (p.A933V). These two patients exhibited spinal disease onset without cognitive impairment, and their onset age and diagnosis delay was within the average range of Chinese ALS patients. Our results suggested that variants in *DCTN1* are not common risk factors for Chinese sporadic ALS and that the frequency of variants of unknown significance in the cohort study was 0.39%.

## Introduction

Amyotrophic lateral sclerosis (ALS) is a devastating neurodegenerative disorder characterized by the selective impairment of the motor cortex and motor neurons of the brainstem and the spinal cord. Approximately 90% of ALS cases are sporadic, and the remaining 10% are inherited[1, 2]. A recent study of 1624 Chinese ALS patients substantially advanced the understanding of the clinical features and epidemiology of this rare disease[3]. Compared with other nationalities, Chinese patients exhibited an earlier age of onset, a lower percentage of bulbar-onset ALS and better prognosis. It is increasingly recognized that ALS represents a heterogeneous group of diseases that share clinical features rather than a single entity [4]. Genetic studies are important for shedding light on the potential mechanisms of heterogeneity associated with ALS, and such studies have been performed worldwide. The human dynactin subunit 1 gene (*DCTN1*) was first identified in a family co-segregating with ALS in 2004[5]. Although *DCTN1* is not a common genetic cause in familial cases, the mutation frequencies in sporadic ALS vary from 0.51% to 2.87% in different populations [6–9]

*DCTN1* is located on chromosome 2p13 and encodes six different isoforms in human. The isoform 1 (known as P150) is a component of the dynactin complex that is expressed in almost every cell[10]. Dynactin subunit 1 includes a CAP-Gly domain, a dynein-binding domain, an Arp1 and Hap1 binding site and they are connected with 3 coiled coil structures (CC1a,CC1b,CC2)[11, 12]. The dynactin complex acts as a connector of cargos with microtubules and cytoplasmic dynein in the process of retrograde vesicle transport, which is involved in centripetal movement of lysosomes and endosomes, ER-to-Golgi transport, spindle formation, chromosome movement, nuclear positioning, and axonogenesis[5, 13]. Disturbing this interaction influences fast axonal transport and consequently disrupts physiologic neuronal function[11]. Mutations of *DCTN1* are associated with distal hereditary motor neuropathy VIIIB (OMIM 607641), Perry syndrome (OMIM 168605) and ALS (OMIM 105400)[5, 12, 14]

At least 14 missense mutations and a splicing change of *DCTN1* were reported in familial and sporadic ALS patients, but the clinical manifestations of ALS caused by mutant *DCTN1* were different among families[5, 7, 15–18]. However, most of the cases were identified in Caucasians. It has been described that the frequencies of mutations of some genes (especially *C9orf72*) in Chinese sporadic ALS was inconsistent with Caucasians[19–21]. In our study, *DCTN1* was first sequenced in a large Chinese population with sporadic ALS, and we wanted to investigate the mutation frequencies and spectrum in *DCTN1*.

## Material and methods

### Ethics statement

This study was approved by the institutional ethics committee of Peking University Third Hospital (PUTH) (IRB00006761). The study group obtained written informed consent from each patient before they participated in the study.

### Participants

This study included 510 sporadic ALS patients (males: females ratio was 1.78; mean age at diagnose ± standard deviation (SD) = 53±11 years) registered with the Neurological Department of Peking University Third Hospital from 2013 to 2014 and 210 non-neurologic control subjects (117 males, 93 females; Males: Females ratio was 1.26; mean age ± SD = 55±13 years). Additional 280 non-neurologic controls (490 controls in all) were used to verify the suspicious pathogenic variants found in patient cohort. All subjects were of Han ethnicity, and the ALS patients met the revised El Escorial criteria of clinically definite, probable, or laboratory-supported probable ALS[22]. Diagnosis delay means the time from symptom onset to a confirmed diagnosis of ALS. All included patients were participating in long-term follow-up and the revised ALS functional rating score (ALSFRS-R) was evaluated every three months prior to study enrollment. We screened *SOD1*, *TARDBP*, *FUS* and *C9orf72* genes for all patients before our research.

### Sequencing analysis of *DCTN1*

Genomic DNA was extracted from whole blood using standard protocols (QIAGEN, Valencia, California, USA). A total of 24 pairs of primers were designed for PCR amplification of the 32 protein-coding exons, including intron-exon boundaries, of *DCTN1* (GenBank NM_004082.4). PCR was performed in a total volume of 25 μl, which consisted of 20 ng of genomic DNA, 10 pmol of each primer, 12.5μl of 2×Taq PCR Master Mix (Tsingke Biotechnology Co., Ltd., Beijing, China) and 9.5μl of deionized water. The thermocycling conditions were as follows: initial denaturation at 98°C for 2 min, 30 cycles at 98°C for 10 s, 57–65°C for 15 s (the annealing temperature ranged from 57°C to 65°C depending on the primer pair, see supplementary material), and extension at 72°C for 15 s, and a final extension at 72°C for 5 min. We used agarose gel electrophoresis and magnetic beads cleanup method for isolating and purifying PCR products. BigDye Terminator v3.1 Cycle Sequencing Kit and 3730XL DNA Analyzer was used for the amplification of PCR products and Sanger sequencing. All products were sequenced at Tsingke Biotechnology Co., Ltd. Each identified mutation was confirmed by both forward and reverse sequencing, and all mutated sequences were re-amplified and re-sequenced.

### Software used to design primers and analyze data

The primers were designed using the Primer3Plus online tool (http://www.primer3plus.com; the primer sequences are available in S1 Table). The sequencing results were analyzed using DNAStarLasergene 7.1 software. The PolyPhen-2 (http://genetics.bwh.harvard.edu/pph2), MutationTaster (http://www.mutationtaster.org), SIFT (http://sift.jcvi.org/) programs were used to predict the effects of each mutation on protein function. The Short Genetic Variations Database (dbSNP 142), the NHLBI Exome Variant Server (EVS), the 1000 Genomes Project (TGP), and the Exome Aggregation Consortium (ExAC) databases were checked for the presence of the variants identified in the cohort. The PhyloP and PhastCons (http://compgen.bscb.cornell.edu/phast/) were used to determine whether the variants were evolutionary conserved. The UniProt Web site (http://www.uniprot.org) was used for the alignment of the mutation sites.

## Results

### Genetic analyses

**Fig 1:**
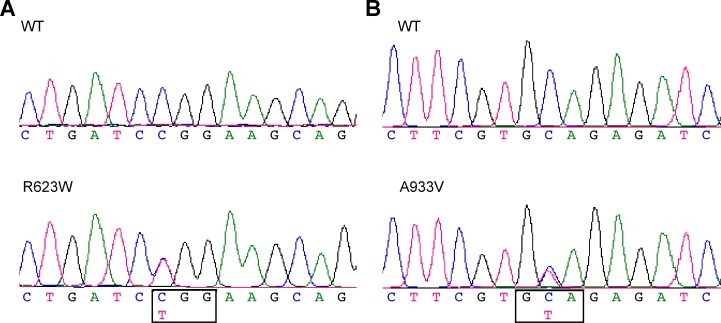
Novel missense mutations in *DCTN1* identified in sporadic ALS patients. (a), (b): Partial chromatograms of two novel mutations of *DCTN1*.

Sequence analysis of *DCTN1* in the patients’ cohort revealed 16 single nucleotide variants (listed in S2 Table). Among them, 14 variants were considered benign polymorphisms, because they were synonymous or have been identified in EVS, TGP or ExAC database (more than 4,300 exomes from East Asian were documented in ExAC). The remaining two variants are heterozygous single base-pair substitutions, c.1867C>T (p.R623W) and c.2798C>T (p.A933V) (GenBank NM_004082.4 and NP_004073.2, see Fig 1 and Table 1). These sites were wild type in all 490 controls. The variants did not affect the splicing site. Moreover, we confirmed the lack of mutations in the *SOD1*, *TARDBP*, *FUS* and *C9orf72* genes in these two patients.

**Table 1:** Novel mutations of *DCTN1* in Chinese patients with sporadic amyotrophic lateral sclerosis.

Mutations	cDNA	Exon	Patients	Controls	MutationTaster	PolyPhen-2 (score)	SIFT (score)	PhyloP (score)	PhastCons (score)
p.R623W	c.1867C>T	17	1/510	0/490	Disease causing	Probably damaging (0.994)	Damaging (0.016)	Conserved (2.479)	Conserved (1)
p.A933V	c.2798C>T	24	1/510	0/490	Disease causing	Probably damaging (0.993)	Damaging (0.018)	Conserved (5.8)	Conserved (1)

**Fig 2:**
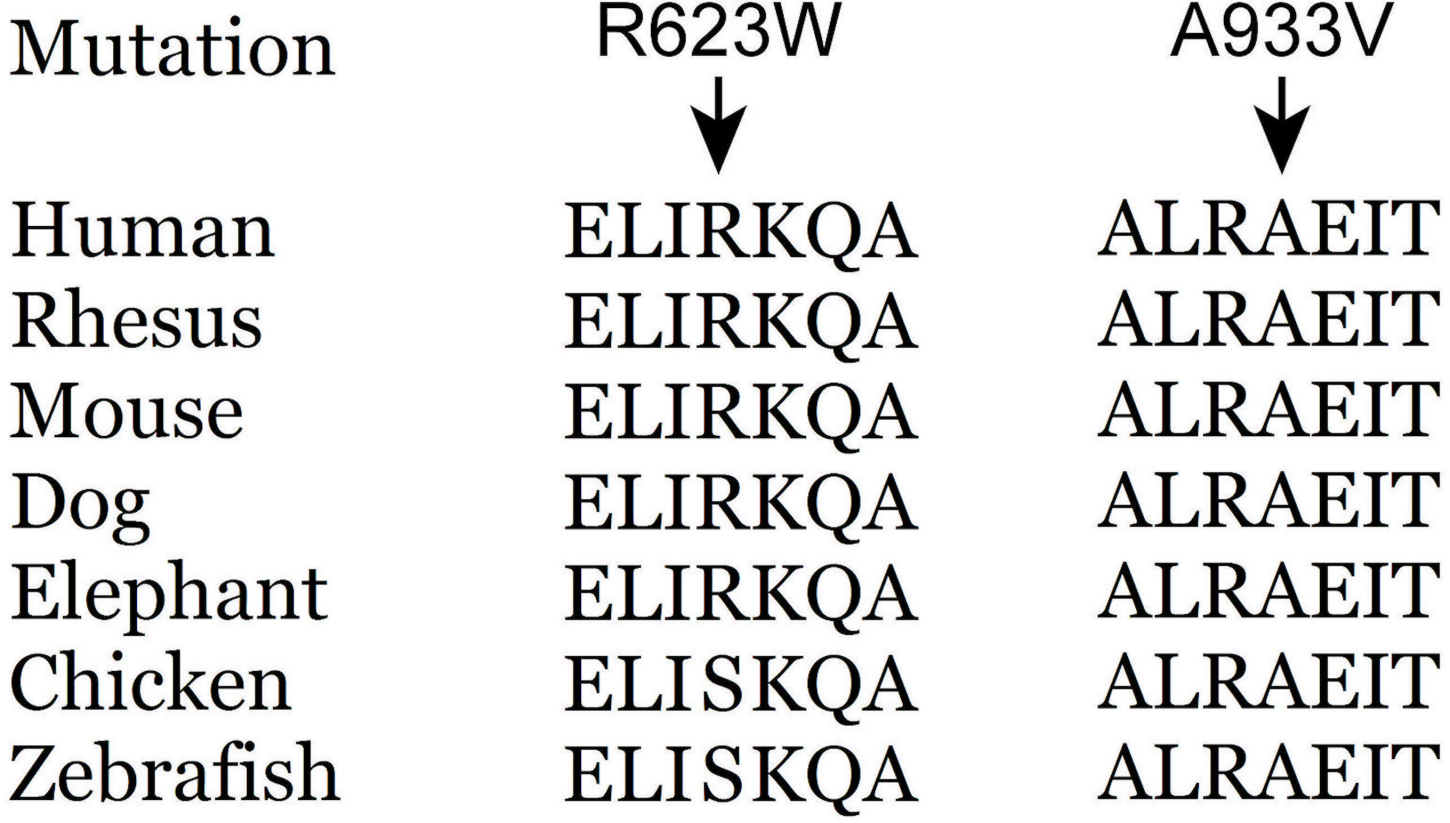
Alignment of human dynactin subunit 1 (isoform 1) amino acid sequences from different species. The p.R623W variant was conserved in mammals, p.A933V variant was conserved in vertebrata.

Supported by PhyloP and PhastCons software (see Table 1), both p.R623W and p.A933V locate in a conserved dynein-binding site, between two coiled coil structures. The alignment of conservation of these sites is shown in Fig 2. According to 3 different programs, p.R623W and p.A933V were considered likely pathogenic (summarized in Table 1).

### Clinical features

The two patients carrying the two variants denied symptoms of dementia or static tremor, they also denied family history of ALS or limb weakness. The cognitive screens on the first visit were insignificant. The follow-up examinations did not show any cognitive impairment or parkinsonian symptoms.

The patient with p.R623W was a female with an ALS onset age of 61 years. The first symptom was weakness of the left hand, then the weakness spread to right hand. 4 months later, both legs were involved. Neither dysarthria nor other bulbar symptoms were observed when she visited the clinic. She had no family history of ALS or other neuromuscular diseases. An EMG study revealed diffuse neurogenic changes, a pathologically brisk deep tendon reflex was observed upon neurologic examination, and Babinski sign was positive for both feet. The diagnosis delay was 6 months, and the FRS-R score at the first visit was 34/48, 2 years after the onset of disease reveals a 23/48 of FRS-R score, the progression rate was 0.46 score per month.

The patient with p.A933V was a 41-year-old female. She complained of right leg weakness without an evident cause. After 8 additional months, she presented with contralateral leg weakness. She developed dysphagia in 9 months after the first symptom. Her symptoms had not fluctuated. She had no family history of neuromuscular disease. On examination, she exhibited weakness and muscle atrophy in all four limbs with occasional spasticity, although the sensory, autonomic, and extrapyramidal systems were normal. EMG showed diffuse neurogenic changes in the sternocleidomastoid, the first dorsal interosseous, the rectus abdominis and the anterior tibia. The diagnosis delay was 18 months, and the first examined FRS-R score was 38/48, 2 years’ follow-up reveals a 30/48 of FRS-R score, the progression rate was 0.33 score per month.

## Discussion

This was the first study assessing *DCTN1* gene in a large sporadic ALS cohort of Chinese mainland. The present study distinguished two novel variants in patients’ cohort, p.R623W and p.A933V, the pathogenicity is supported by the following facts. These two variants were not present in the 490 race-matched control or population based databases (EVS, dbSNP, 1000 Genomes Project, and ExAC, including more than 65,000 individuals, >4,300 East Asian population) and both located in a highly-conserved region. The pathogenicity is also supported by *in silico* prediction, all 3 software suggest a deleterious effect of the mutant gene. Although we lack the parental confirmation for these two variants but they denied familial history, so these variants could be potential *de novo* mutations. According to the ACMG guideline[23], the pathogenicity for these two variants were supported by evidence PM2 and PP3, so these two would be judged as variants of uncertain significance (VOUS). Another variants p.G1062D (c.3185G>A) discovered in our patient cohort was also reported in a Japanese ALS cohort[9]. But the variant was observed in East Asian population with a low frequency (MAF = 0.0001156) from the ExAC database. Further functional study will be contributive.

The present study distinguished two novel VOUS. The frequency of *DCTN1* rare variants in sporadic ALS was 0.39% (2 of 510), lower than the data previously reported in sporadic ALS in Japanese (1.07%, 5 of 469)[9], American (2.07%-2.87%, 5 of 242, 10 of 349)[6, 7]and Irish (0.51%, 2 of 394)[8]. Although the size of control cohort and reference genetic databases used were different between studies, our result could also be a reflection of different frequency or penetrance of *DCTN1* gene between populations. It is reported that rare variants of *DCTN1* gene may coexist with variants of other ALS causing genes (*SETX*, *ANG*, *SOD1*, *FIG4*)[6]. So, there was also a possibility that oligogenic mutations may be a factor for our variant carriers. This observation suggests genetic diversity and provides an opportunity to explore the mechanisms of rare variants related to ALS. Our patients carrying *DCTN1* VOUS did not reveal a uniform clinical manifestation, partly because number of ALS patients carrying such variants was too few to establish any characteristic.

p150 protein contains a shoulder and 3 coiled-coil structures (CC1a, CC1b, CC2) of the dynactin complex[24]. The highly-conserved GAP-Gly domain of p150 connects the microtubules via residues 29–95 near the N-terminus[11]. Mutations in CAP-Gly domain of the dynactin may cause Parkinson’s disease with hypoventilation and depression (Perry syndrome)[14]. The p.R623W and p.A933V variants located in the dynein-binding domain, several ALS-causative mutations (p.M571T, p.H668I and p.R785W) are in this domain[5, 7]. A defective ICD domain may disrupt the dynein-dynactin complex to transport cargos along microtubules and this will affect the axonal transport, which plays a vital role in motor neuron diseases[25, 26]. Interestingly, mutations of other dynactin related protein such as protein bicaudal D homolog 2(gene *BICD2*), and cytoplasmic dynein heavy chain (gene *DYNC1H1*) may cause lower extremity-predominant spinal muscular atrophy (SMALED1, OMIM 158600, and SMALED2, OMIM615290),which affects the lower motor neuron in spinal cord. It indicates that the dynein-dynactin complex is important for motor neuron survival.

In conclusion, we assess the frequency of *DCTN1* variants in a Chinese cohort of sporadic ALS cases and controls. We identified two novel missense VOUSs, p.R623W and p.A933V, the frequency was 0.39% for Chinese sporadic ALS. Our results suggest variants in *DCTN1* are not common risk factors for Chinese sporadic ALS patients.

## Supporting information

S1 Table 24 primers of 32 coding exons in *DCTN1*. (DOCX)

S2 Table *DCTN1* single nucleotide variants in sporadic ALS cohort. (DOCX)
