# Supplementary material for: DCTN1 gene analysis in Chinese patients with sporadic amyotrophic lateral sclerosis
Source: PLoS One. 2017 Aug 8;12(8):e0182572. doi: 10.1371/journal.pone.0182572 (PMC5549744; doi:10.1371/journal.pone.0182572)
Supplement: S1 Table — (DOCX) [file pone.0182572.s001.docx]

# S1 Table. 24 primers of 32 coding exons in *DCTN1*

|  | FORWARD | REVERSE | CDS | Annealing temperature (°C) |
| --- | --- | --- | --- | --- |
| 1 | CCTTAGTTTCTGGCCCACGT | AAGCCTGTCCAGCCTTACAC | 1 | 61 |
| 2 | AGCATCTTGGCTGAGGCATT | AACCAAGAGTACAGGGCAGC | 2 | 61 |
| 3 | GAGGGGCCCATATTACAGCC | GACCCTGAACTTCTGGAGCC | 3&4 | 63 |
| 4 | TGGCCCTAGTAGTGTAGCCA | CCACCATTTCCTCTGGCCAT | 5 | 61 |
| 5 | GGAGGCTGAGACTGCATTGT | TACCACAGGAAACCCTCCCA | 6 | 63 |
| 6 | CTGGCTCTGGACTGGAGTTG | AAGCCATTTTCCCCAGGAGG | 7 | 65 |
| 7 | GGAATGTGGAGTCAGTGCCA | TAGGTCTTTGCCAACCCCAG | 8 | 63 |
| 8 | TATGCCACTGACTGTTGGCC | TAGCGTTCCTTTGCCTCCAG | 9 | 63 |
| 9 | CTAGACTGGAGCTGGAGCCT | AGAAAGATCCCGCATCCTGC | 10&11 | 65 |
| 10 | TGTGTTAGAGCACCCATCGC | AAAAGGCAGGGTCAGGGTAG | 12&13 | 65 |
| 11 | CCCTGCCTTTCTTTAGCCCA | AATTCCCATGCCTTGCTCCA | 14&15 | 65 |
| 12 | GGGATCTACTGGAGCAAGGC | GCTATCCTGCCCATCATCCC | 16 | 63 |
| 13 | CTCCCATGTGTGTCCATGCT | AAGAGCAAACCAGGCCTCAA | 17&18 | 61 |
| 14 | CAAGAATTTGGGCTTGGGGC | ACTCTGCGTGAACTGTGAGG | 19 | 63 |
| 15 | AGCAGAGGATGGGGACTTCT | GCCAGAGTCAGGAGTCAACC | 20&21 | 63 |
| 16 | TCTGGATCTGGTTAGGGGACA | TCTAGGCAGGATGGTGCTCT | 22&23 | 61 |
| 17 | GAGGAAGGCATCTGGAGAGC | ATGCGCTCATCTGCATCCTT | 24 | 63 |
| 18 | TCACAGATGCTGAAGGCCTG | GGGTTGGCAGTGGGTAAAGA | 25 | 61 |
| 19 | GCAGGGCTATTCCAAGGACC | ATGAGGCCAAGGACAGGAGA | 26 | 61 |
| 20 | CCAGGATTCCTCTGGTGTGT | GGCCCCTGTCATCTATCATCA | 27&28 | 61 |
| 21 | GGGCCTCCTAGCACCTCTTA | ACACTGCTGGGAAGAGCTTC | 29 | 65 |
| 22 | CCTGCACCCCTAACCCTTAA | TCTCCTCCCCAATTGCTGTC | 30 | 57 |
| 23 | TGGTCTTGTGCTAGCCTGTG | GACCTGAGACTCCAAAGGCC | 31 | 61 |
| 24 | TGTGGTCACAATAGCCCAGG | GGCAGGAAGAGCTTAACCCA | 32 | 61 |
