# Supplementary material for: DCTN1 gene analysis in Chinese patients with sporadic amyotrophic lateral sclerosis
Source: PLoS One. 2017 Aug 8;12(8):e0182572. doi: 10.1371/journal.pone.0182572 (PMC5549744; doi:10.1371/journal.pone.0182572)
Supplement: S2 Table — (DOCX) [file pone.0182572.s002.docx]

# S2 Table. *DCTN1* single nucleotide variants in sporadic ALS cohort

| bp change | variants | type | location | exon | sALS | Control | dbSNP | TGP | ExAC |
| --- | --- | --- | --- | --- | --- | --- | --- | --- | --- |
| c.213A>C | p.Gly71Gly | synonymous | 2:74605193 | 2 | 2/510 | 0 | NA | 0 | 5 |
| c.419C>T | p.Pro140Leu | missense | 2:74601463 | 6 | 1/510 | 0 | rs147939455 | 0 | 1 |
| c.1268T>C | p.Ile423Thr | missense | 2:74597332 | 12 | 1/510 | 0 | rs201127181 | 1 | 0 |
| c.1822G>A | p.Val608Met | missense | 2:74595887 | 16 | 1/510 | 0 | NA | 0 | 12 |
| c.1867C>T | p.Arg623Trp | missense | 2:74595246 | 17 | 1/510 | 0 | NA | 0 | 0 |
| c.2213A>G | p.Gln738Arg | missense | 2:74594519 | 19 | 2/510 | 2 | rs143800457 | 7 | 56 |
| c.2448A>G | p.Ala816Ala | synonymous | 2:74593928 | 21 | 1/510 | 0 | rs1130484 | 80 | 1325 |
| c.2798C>T | p.Ala933Val | missense | 2:74593108 | 24 | 1/510 | 0 | NA | 0 | 0 |
| c.2909A>G | p.Asn970Ser | missense | 2:74592762 | 25 | 1/510 | 0 | rs568812456 | 0 | 1 |
| c.3145C>T | p.Arg1049Gln | missense | 2:74592253 | 26 | 1/510 | 0 | rs573012389 | 1 | 2 |
| c.3185G>A | p.Gly1062Asp | missense | 2:74592213 | 26 | 1/510 | 0 | NA | 0 | 1 |
| c.3292T>G | p.Ser1098Ala | missense | 2:74590474 | 28 | 1/510 | 0 | NA | 0 | 1 |
| c.3490A>G | p.Thr1164Ala | missense | 2:74590160 | 29 | 3/510 | 0 | rs72466493 | 18 | 63 |
| c.3721G>A | p.Asp1241Asn | missense | 2:74588742 | 32 | 2/510 | 1 | rs184429549 | 2 | 18 |
| c.3760G>T | p.Ala1254Ser | missense | 2:74588703 | 32 | 2/510 | 0 | NA | 0 | 12 |
| c.3799G>C | p.Glu1267Gln | missense | 2:74588664 | 32 | 6/510 | 1 | rs146083590 | 0 | 16 |

dbSNP: The RS number of the variant in Short Genetic Variations Database(http://www.ncbi.nlm.nih.gov/snp)

TGP: The number of carrier of the variant in 1000 Genomes Project(http://browser.1000genomes.org)

ExAC: The global number of carrier of the variant in Exome Aggregation Consortium(http://exac.broadinstitute.org/)
